# The role of subcutaneous adipose tissue in supporting the copper balance in rats with a chronic deficiency in holo-ceruloplasmin

**DOI:** 10.1371/journal.pone.0175214

**Published:** 2017-04-05

**Authors:** Ekaterina Y. Ilyechova, Nadezhda V. Tsymbalenko, Ludmila V. Puchkova

**Affiliations:** 1 Department of Molecular Genetics, Institute of Experimental Medicine, St. Petersburg, Russia; 2 International Research and Education Center "Functional materials and devices of optoelectronics and microelectronics", ITMO University, St. Petersburg, Russia; 3 Department of Biophysics, Institute of Physics, Nanotechnology, and Telecommunications, Peter the Great St. Petersburg Polytechnic University, St. Petersburg, Russia; Karolinska Institutet, SWEDEN

## Abstract

We have previously shown that (*1*) an acute deficiency in blood serum holo-ceruloplasmin (Cp) developed in rats that were fed fodder containing silver ions (Ag-fodder) for one month and (*2*) the deficiency in holo-Cp was compensated by non-hepatic holo-Cp synthesis in rats that were chronically fed Ag-fodder for 6 months (Ag-rats). The purpose of the present study is to identify the organ(s) that compensate for the hepatic holo-Cp deficiency in the circulation. This study was performed on rats that were fed Ag-fodder (40 mg Ag·kg^-1^ body mass daily) for 6 months. The relative expression levels of the genes responsible for copper status were measured by RT-PCR. The *in vitro* synthesis and secretion of [^14^C]Cp were analyzed using a metabolic labeling approach. Oxidase activity was determined using a gel assay with *o-*dianisidine. Copper status and some hematological indexes were measured. Differential centrifugation, immunoblotting, immunoelectrophoresis, and atomic absorption spectrometry were included in the investigation. In the Ag-rats, silver accumulation was tissue-specific. Skeletal muscles and internal (IAT) and subcutaneous (SAT) adipose tissues did not accumulate silver significantly. In SAT, the mRNAs for the soluble and glycosylphosphatidylinositol-anchored ceruloplasmin isoforms were expressed, and their relative levels were increased two-fold in the Ag-rats. In parallel, the levels of the genes responsible for Cp metallation (*Ctr1* and *Atp7a/b*) increased correspondingly. In the SAT of the Ag-rats, Cp oxidase activity was observed in the Golgi complex and plasma membrane. Moreover, full-length [^14^C]Cp polypeptides were released into the medium by slices of SAT. The possibilities that SAT is part of a system that controls the copper balance in mammals, and it plays a significant role in supporting copper homeostasis throughout the body are discussed.

## Introduction

The biological role of copper as a catalytic and structural cofactor of vitally important enzymes and as a potentially toxic agent was established more than half a century ago [1,2]. In the last 20 years, the system of safe copper transport from extracellular spaces to the places where cuproenzymes are formed was discovered and studied in detail [3]. In the last 5 years, it has become evident that copper participates in the regulation of proliferation, apoptosis, neovascularization, neurotransmission, and signaling; thus, it may be viewed as a secondary messenger, somewhat similar to calcium [4–8]. Disturbances in copper homeostasis lead to the development of cardiovascular, neurodegenerative, and oncological diseases [2,7,9]. The attention of researchers has mainly been focused on intracellular copper homeodynamics. However, homeostasis and the maintenance of copper balance in body fluids are insufficiently studied.

The extracellular copper balance is characterized by copper status indexes. The term “copper status” typically denotes a set of blood serum indexes: copper concentration, immunoreactive ceruloplasmin (Cp) protein content and oxidase activity (the level of holo-Cp) [10]. Cp is a multicopper blue (ferr)oxidase of vertebrates, and it accounts for up to 95% of extracellular copper in mammals [11]. It’s the major biological role is conversion of Fe(II) to Fe(III) to iron transport through membranes [12]. According to the acknowledged paradigm, the mammalian liver plays a central role in copper turnover in the body [3]. Copper, which is absorbed in the small intestine, is captured from the bloodstream by hepatocytes; in these cells, copper is inserted into Cp, which is secreted into the bloodstream. It has been shown that there is a mutual dependence on copper status and copper metabolism in the cells of various organs [9,13]. Recently, some evidence suggested that inter-organ copper-mediated communication exists and regulates copper metabolism in the liver, based on the current requirements of the extra-hepatic organs. Therefore, the tissue-specific deletion of high affinity copper transporter gene *CTR1*, which produces a copper deficiency in heart cells, also induces the liberation of copper from the liver [14]. Additionally, the liver copper metabolism is stimulated by growing tumors, which are severely impaired in the absence of hepatic holo-Cp [15,16].

**Fig 1:**
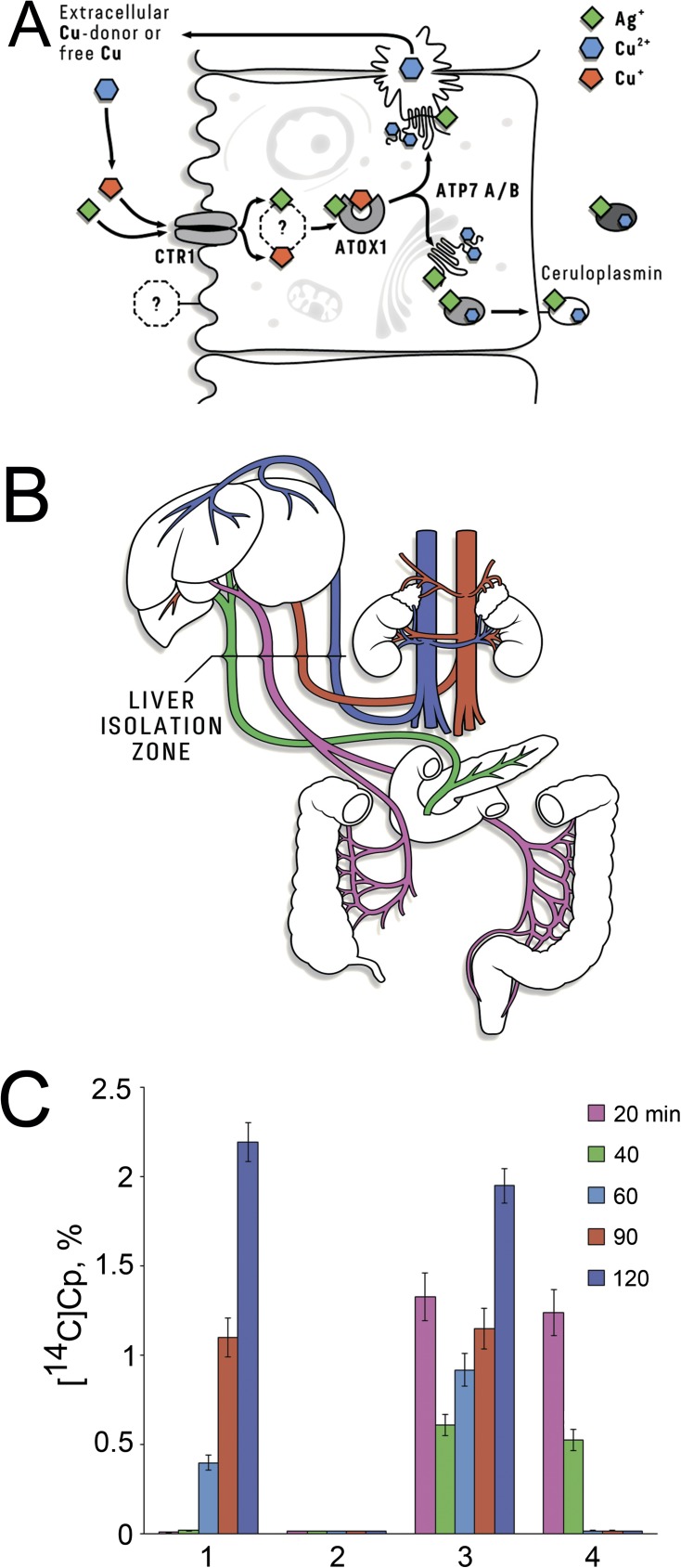
The explanation of why non-hepatic ceruloplasmin (Cp) is thought to be found in the blood of Ag-rats. **(A)** Schema of Cu(I)/Ag(I) moving in liver cells [17]. The Cu(I)/Ag(I) ions that are absorbed in the small intestine are delivered to the liver, transferred by CTR1 through the hepatocyte membrane, bound to the cytosolic Cu(I)/Ag(I)-chaperon (ATOX1), and transmitted to copper-transporting P1-type ATPases (ATP7A/B) that are integrated into the trans-Golgi membrane. The last transfers move Cu(I)/Ag(I) ions to the Golgi lumen and includes them in the Cp (dark circle). The Ag-Cp molecules lost enzymatic activity and the holo-Cp level was reduced (light circle). **(B)** The surgery used to isolate the liver from the circulation. The surgery was performed with the ligature of the common bile duct, the portal vein and the hepatic artery. In addition, the left carotid artery was catheterized using a catheter with a plug at the distal end, through which [^14^C] amino acids were injected and blood samples were subsequently taken over different time intervals. **(C)** Dynamics of appearance of *de novo* synthesized [^14^C]sCp in the blood serum in pulse-experiments. Abscissa: 1 –control rats, [^14^C]sCp appeared in 60 min after injected [^14^C] amino acids and reached a plateau in 2 h; 2 –in intact rats with liver isolated from circulation, [^14^C]sCp did not appear; 3 –in Ag-rats, [^14^C]sCp appeared as two portions: in 20 min and 90 min; 4 –in Ag-rats with livers isolated from the bloodstream, [^14^C]sCp came in 20 min after start pulse-experiment. Ordinate: [^14^C]Cp, %.

In our previous work, we showed that hepatic holo-Cp production can be affected by silver ions in fodder (Ag-fodder) [17]. This occurs because Ag(I) and Cu(I) are isoelectronic; therefore, Ag(I) ions are bound by copper transporters and erroneously substitute for copper in the Cp molecule (Fig 1A). Adult rats that were maintained on the Ag-fodder for one month displayed a decrease in the serum copper and holo-Cp concentrations to approximately zero. In contrast, in the rats that received the Ag-fodder from an early postnatal period for 6 months, the copper concentration and oxidase activity in the bloodstream were ~50% of the typical physiological values (Table 1). The pulse-chase experiments on the intact rats with liver isolated from the bloodstream (scheme in Fig 1B) showed that radiolabelled Cp did not appear in the bloodstream, although it was detected in the rat with no impaired circulation (Fig 1C). In the Ag-rats with liver isolated from the bloodstream, radiolabelled Cp was detected (Fig 1C). This observation can be explained by the fact that chronic deficiency of holo-Cp of hepatic origin is compensated by ectopic synthesis of this protein (Fig 1).

**Table 1:** The some physiological and biochemical indicators for rats chronically fed with Ag-diet.

PARAMETERS	Animal group		
	Intact rats	Short time Ag-diet	Chronic Ag-diet
*Serum copper status*	n = 10	n = 15	n = 20
[Cu], μg·l^-1^ (n = 10)	1306 ± 100	120 ± 10*	981 ± 28*
[Ag], μg·l^-1^ (n = 10)	not assessed	2050 ± 210	1580 ± 240
[Cp] protein, g·l^-1^, (n = 8) ^a^	65 ± 7	58 ± 5 (NS)	60 ± 8 (NS)
Oxidase activity, g·l^-1^, (n = 10) ^b^	38.5 ± 3.4	1.7 ± 0.5*	20.0 ± 0.7*
Ferroxidase activity, a.u., (*n* = 5)^c^	1.0 ± 0.23	0.028 ± 0.01*	0.70 ± 0.14*
Hemoglobin, g·l^-1^, (*n* = 5)	172 ± 21	168 ± 15 (*NS*)	175 ± 30 (*NS*)

^a^values were determined by rocket immunoelectrophoresis;

^b^values were determined by coloremitric method with *p*-phenylenediamine;

^c^values were determined by gel-assay.

*P < 0.05.

The aim of the present study was to identify the organ(s) that compensate for holo-Cp production in Ag-rats, which will help extend our knowledge of copper metabolism in the mammalian body.

## Materials and methods

### Animals and their treatment

In this study, 2-month-old, Wistar rats were purchased from Rappolovo nursery (Leningrad Region, Russia) to obtain newborns in the vivarium of the Research Institute of Experimental Medicine. Groups of 10 juvenile rats or fewer adult animals or a female with a litter (eight newborns in a litter) were housed in plastic cages (1815 cm^2^ and 720 cm^2^, respectively) with wood cutting waste. The animals were housed with a 12:12-h light-dark cycle and ∼60% humidity at 22–24°C and were given suitable fodder and water *ad libitum*. Standard fodder was purchased from LTD^Co^ “Fodder for laboratory animals” (Moscow, Russia). All experiments were carried out on males.

Procedures involving animals and their care were conducted in conformity with institutional guidelines and are in compliance with national laws (Russian Federation the Ministry of Health N267, June 19, 2003; Guide for the Use of Laboratory Animals, Moscow, 2005). The studies were approved by the local Committee of Ethics at the Institute of Experimental Medicine (Protocol number N2/13 was approved on 27 June 2013, Pavlov str., 12, St. Petersburg, 197376 Russia).

The rats were sedated with diethyl ether vapor and euthanized by cervical dislocation, which was performed by skilled personnel. The animals that were used to isolate the subcellular fractions or metabolic syntheses were anesthetized with sodium oxybutyrate (0.1 g·kg^-1^ of body weight).

Ag-rats were fed by females that received Ag-fodder beginning on the first day of lactation. The weaned pups were fed Ag-fodder. The preparation of the fodder was described previously [17]. Briefly, 330 mg of silver (from AgCl) were added to 1 kg of standard fodder and the fodder was very thoroughly mixed and moistened with distilled water (∼ 1:1 w/v). Supposing that a rat (about 250 g body weight) eats about 30 g dry fodder daily, and it was estimated that the rats consumed approximately 50 mg AgCl·kg^-1^ body weight daily. The rats were analyzed at the age of 6 months. The reference group consisted of rats that were born at the same time and housed in the same conditions as the experimental group but received standard fodder.

### Isolation of fractions enriched with plasma and Golgi complex membranes

The fractions enriched with plasma and Golgi complex membranes were isolated by differential centrifugation. The tissue samples were homogenized (1:9 w/v, respectively) 3×20 s in buffer A containing 250 mM sucrose, 100 mM KCl, 5 mM MgCl_2_, 10 mM Tris-HCl (pH 7.4), 5 mM DTT, and 0.5 μl·ml^-1^ protease inhibitor cocktail (Sigma, USA) using a T10 basic homogenizer (IKA, Germany) at maximum power. The homogenate was centrifuged at 800×g for 10 min. The resulting pellet consisted of nuclei and large plasma membrane fragments, which contained ~80% of the total cellular ouabain-sensitive Na/K-ATPase activity [18]. The 800×g pellet was resuspended in buffer A, loaded onto a 1.5 M sucrose cushion, and centrifuged at 15000×g for 4 h in a basket rotor. The material located above the sucrose cushion was collected, diluted with buffer A (without sucrose), collected again by centrifugation at 1000×g for 30 min, and resuspended in buffer A to be used as the plasma membrane-enriched fraction. The 800×g supernatant was centrifuged at 12000×g for 20 min to separate the mitochondria, lysosomes and peroxisomes. The resulting supernatant was centrifuged (23000×g for 1 h) to sediment the Golgi complex membranes. The pellet was resuspended with buffer A and used as the Golgi complex-enriched fraction. Both fractions were incubated with Triton X-100 (final concentration 1%) for 30 min at 0°C, clarified by centrifugation at 15000×g for 1 h and used to determine the holo-Cp and Cp protein levels. The method for plasma membrane and Golgi complex isolation did not exclude the slight contamination with other cell fractions (*e*.*g*. microsomes, early endosomes, primary lysosomes, *etc*.). Nevertheless, they can be used because none of these cell fractions contains a full-length oxidase positive Cp.

### Synthesis of [^14^C] soluble ceruloplasmin (sCp) in the subcutaneous adipose tissue sections and immunoprecipitation

The samples (approximately 200 mg) of subcutaneous adipose tissue (SAT) were cut into pieces and rinsed with PBS. The washing buffer was removed and the sections were incubated with 1.5 ml of DMEM containing a [^14^C]-amino acid mixture solution (100 μCi·ml^-1^, Amersham, UK) in wide bottom flasks (the liquid layer did not exceed 3 mm) for 90 min at 37°C with moderate shaking. After incubation, the pieces were separated by centrifugation (2000×g for 10 min at 4°C). The supernatant was collected and 0.5 μl·ml^-1^ protease inhibitor cocktail was added. Then, the supernatant was clarified by further centrifugation (15000×g for 60 min), and the resulting supernatant was used to precipitate the sCp. The tissue pieces were washed with DMEM, centrifuged again, and then homogenized in buffer A to isolate the plasma membrane and Golgi complex membranes, and Triton X-100 extracts were obtained as described above. For immunoprecipitation, rat serum aliquots (6 μl), which were used as a carrier of Cp, and 0.5 ml of IgG (10 mg·ml^-1^), which was isolated by salting out by ammonium sulfate from the blood serum of rabbits that were immunized with highly purified rat holo-Cp (A_610/280_ = 0.054) [19], were added to aliquots (approximately 4 ml) of incubation medium and the Triton X-100 extracts. These mixtures were incubated overnight at +4°C. The precipitates were collected, washed twice with PBS, dissolved in electrophoretic sample buffer, and fractionated by 8% PAGE. After PAGE, the proteins were transferred to Protran® nitrocellulose membranes (Sigma-Aldrich, USA). Transfer efficiency was controlled by Ponceau S (Sigma-Aldrich, USA) staining; the nitrocellulose membrane was dried and used for autoradiography (UltraCruz™ film, Santa Cruz Biotechnology, USA). The radioactivity of the total protein fractions and radioactivity of the immunoprecipitates were used to calculate the percentage of newly formed Cp. The experiment was repeated twice.

### Measurement of the relative levels of mRNAs

Total RNA was isolated using TRIzol Reagent (Invitrogen, UK). RNA concentration was measured using a NanoDrop 2000 spectrophotometer (Thermo Scientific, USA) following the standard procedure. The purity of RNA samples was proved by the optical density ratio A_260_/A_280_>1.8. To verify the integrity of the samples, the 18S/28S RNA ratio was analyzed after electrophoresis in 1.4% agarose gel. Design of primers was performed using the Primer-BLAST software (NCBI, USA); the primer sequences, sizes of PCR products and annealing temperatures were presented in Table 2. For each pair of primers the concentrations of primers and MgCl_2_, annealing temperature, and time setup as well as appropriate number of cycles for semi-quantitative PCR were optimized using MJ Mini Personal Thermal Cycler (BioRad, USA). As a result, 25 pM of each primer and 3 mM MgCl_2_ were used for all amplifications. β-actin was selected as the internal control. PCR consisted of the following steps: initial denaturation (5 min at 94°C), cycles of amplification (denaturation– 1 min at 94°C, annealing of primers– 1 min, elongation– 1 min at 72°C) and terminal elongation (7 min at 72°C). Amplification included 28 cycles for β-actin and 30 cycles for others cDNA’s. The electrophoretic analysis of the PCR products demonstrated that their sizes corresponded to the calculated values and non-specific products were not synthesized under the chosen experimental conditions. In all the experiments results were extrapolated on the exponential growth curve. RT-PCR products were analyzed in a 1.4% agarose gel with ethidium bromide and the data processed using ImageJ software. The results were expressed in arbitrary units (a. u.) as a ratio between the amount of the PCR product of the mRNA specified and the amount of the PCR product of β-actin obtained with the same RNA preparations under similar conditions.

**Table 2:** Sequences of primers used for RT-PCR analysis.

Gene	Nucleotide sequence (5'→3') of primers	Product size, bp	T
*Atp7a*	F: gaa gcc tac ttt ccc ggc tac aac aga agc; R: agg tac cca agg ttt cag tgt cca gct cc	421	64
*Atp7b*	F: cag aag tac ttt cct agc cct agc cct agc aag c; R: ccc acc aca gcc aga acc ttc ctg ag	332	65
*β-actin*	F: gaa gat cct gac cga gcg tg; R: agc act gtg ttg gca tag ag	327	59
*Cp*	F:agt aaa caa agt cac aac gag gaa t; R:tcg tat tcc act tat cac caa ttt a	398	57
*GPI-Cp*	F:agt aaa caa agt cac aac gag gaa t; R: ctc ctt ggt aga tat ttg gaa taa a	436	57
*Dmt1*	F: tga gtt ctc caa cgg aat agg ct; R: tga gtt ctc caa cgg aat agg ct	251	60
*Slc31a1 (Ctr1)*	F: tgc cta tga cct tct act ttg g; R: atg aag atg agc atg agg aag	358	57

The gene names are given according to rat genome databases in alphabetical order. F–forward, R–reverse. T–annealing temperature, °C.

### Immunoblotting (WB)

For WB analysis, the samples were equalized regarding protein content, and electrophoresis was performed on 8% polyacrylamide gels (PAGE) with or without 0.1% SDS according to the Laemmli method. The protein transfer, control for the quality and uniformity of transfer with Ponceau S staining, blocking with 5% non-fat milk, blotting with primary rabbit antibodies against rat Cp, and visualization of the immune complexes were described previously [20]. The film was processed using the method reported by Aldridge et al [21]. Hybond ECL nitrocellulose membrane, ECL reagent, ECL Hyperfilm (GE Healthcare, USA), and horseradish peroxidase-conjugated goat anti-rabbit secondary antibodies (Abcam, UK) were used for the WB analysis. In the work, non-commercial antibodies to high purity rat Cp were used [19]. In the rat serum, the antibodies reacted with Cp only as shown Western blot and immunoelectrophoresis [20]. Protein markers with molecular masses ranging from 14.4 to 116 kDa were applied (ThermoScientific, USA).

### Other methods

The oxidase and ferroxidase activities of Cp were revealed by an in-gel assay. After non-denaturating 8% PAGE, the gels were stained with *o*-dianisidine [22]. The total protein concentration was determined by the Bradford assay using BSA as a calibration standard. The atomic metal concentrations were measured with a graphite furnace AAS using a Zeeman correction of non-selective absorption in a ZEEnit 650P spectrometer (AnalytikJena, Germany). The samples were homogenized in PBS and then dissolved in pure HNO_3_. All solutions were prepared in deionized water that had been pretreated with Chelex-100 resin.

The data are presented as averages ± standard deviation. The significance of changes was determined by unpaired two-tailed Student’s *t*-test; the changes were considered significant at *p* < 0.05.

The reagents used for protein and nucleic acid electrophoresis and the salts were purchased from Sigma-Aldrich (USA) and Merck (Germany).

## Results

**Fig 2:**
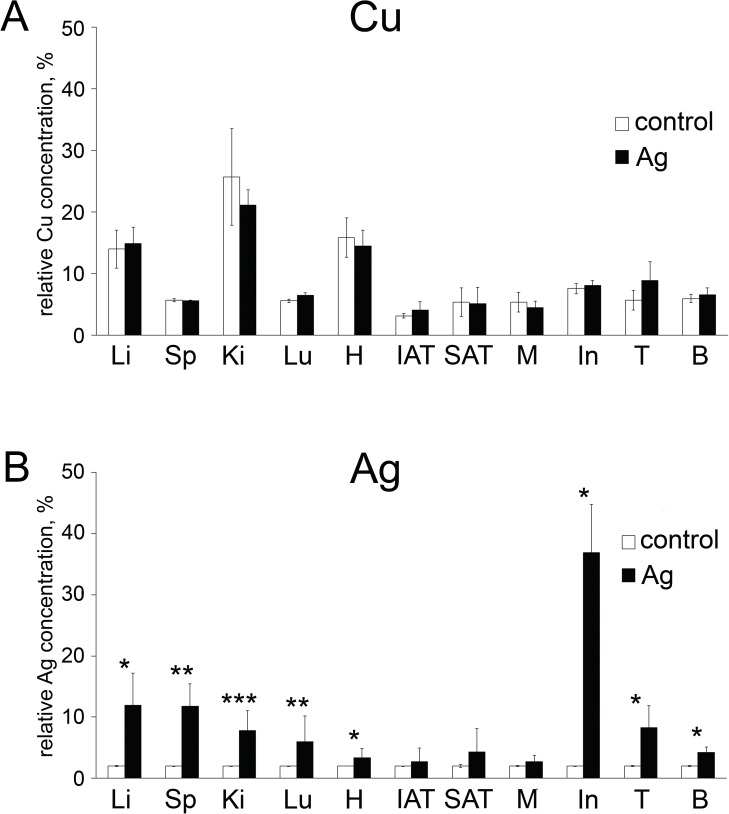
**Distribution of copper (A) and silver (B) in the rats with chronic serum holo-Cp deficiency (*n =* 5).** Ordinate: relative metal concentration, %. Abbreviations: Li–liver, Sp–spleen, Ki–kidney, Lu–lung, H–heart, IAT–internal adipose tissue, SAT–subcutaneous adipose tissue, M–skeletal muscles, In–intestine, B—brain. White bar–control; dark bar–Ag-rats. Ordinate: relative metal concentration, %; the difference was significant at the *: *P* < 0.05, **: *P* < 0.01, and ***: *P* < 0.005 levels.

An organ that could compensate for the deficiency in hepatic holo-Cp in the Ag-rats should meet at least the following two criteria: (1) it does not accumulate silver effectively so silver is not included in holo-Cp (Fig 1A), and (2) it expresses the *Cp* gene. Therefore, in the first stage of the study, the copper concentrations and the silver distributions in the bodies of the Ag-rats were measured. The rats of the same age that were fed standard fodder were used as the reference group. The results are presented in Fig 2. It can be seen that no cellular copper deficiency developed in the Ag-rats (Fig 2A). In general, the relative copper content in the organs agreed with the reported data [23]. The silver accumulation in the rat body was non-uniform (Fig 2B). As expected, the largest amounts of silver were detected in the cells of the small intestine, which acted as a barrier between the Ag-containing fodder and the body environment. The organs were arranged by decreasing silver storage capacity: liver, spleen, testis, kidney, lung, brain, heart and others, which accumulate silver at the background level (skeletal muscles, internal adipose tissue (IAT) and SAT).

Because the primary transcript of *Cp* gene can be processed to two forms of Cp-mRNA by alternative splicing, the Cp-mRNA encoding sCp, and an mRNA encoding membrane-anchored GPI-Cp [24], both isoforms of Cp-mRNA were tested by semi-quantitative RT-PCR. Although both isoforms of Cp are synthesized in brain and testes [24,25], both organs were excluded from the study because the they are separated from the blood by the barriers. The probability of the heart and skeletal muscles serving as the blood holo-Cp sources was assumed to be very low because they do not express the *Cp* gene [26].

**Fig 3:**
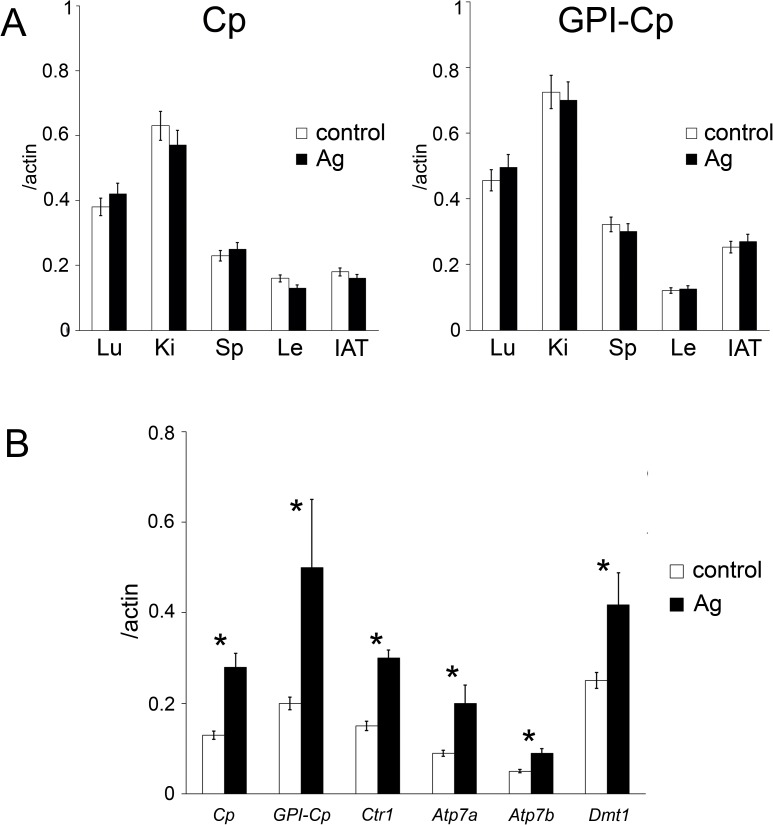
Extrahepatic expression of the *Cp* gene. (**A**) The relative level of Cp and GPI-Cp mRNA (level of Cp(GPI-Cp)/actin, a.u.) in organs from control and Ag-rats (*n =* 5); abbreviations: Lu–lung, Ki–kidney, Sp–spleen, Le–leukocytes, IAT–internal adipose tissue. White bar–control; dark bar–Ag-rats. (**B**) The expression level of genes related to Cp/GPI-Cp metallation in SAT (*n =* 5). Ordinate: relative content of mature transcripts, gene of interest/actin, a.u. White bar–control; dark bar–Ag-rats. *: the difference was significant at the *P* < 0.05 level.

The lungs, kidneys, spleen, IAT, and SAT were tested for the ability to activate *Cp* gene expression under conditions of holo-Cp deficiency. Leukocytes were also included in the study as they synthesized both sCp and GPI-Cp [27]; silver accumulation was not measured in these cells because they are short-lived cells. The data presented in Fig 3A showed that the kidney, lungs, spleen, leukocytes, IAT, and SAT produced both Cp-mRNA forms. However, the Ag-fodder did not stimulate *Cp* gene activity in any of the organs, except SAT (Fig 3B). Moreover, in SAT, the expression of the genes that are involved in the metallation of Cp, i.e., *Ctr1* (encodes universal high affinity Cu(I) importer) [28], *Atp7a* and *Atp7b* (encode Cu(I)/Cu(II)-transporting P1 type ATPases [29] that supply copper atoms to GPI-Cp and sCp, respectively), was significantly increased (Fig 3B). In addition, the expression level of the *Dmt1* gene (encodes divalent metal transporter) [30] was increased by a factor of two. Thus, the expression of the genes that are typically responsible for blood copper status was increased in SAT cells of the Ag-rats. These data allowed us to hypothesize that SAT was a suitable candidate (or one of the candidates) organ that could compensate for the hepatic holo-Cp deficiency in the Ag-rats.

**Fig 4:**
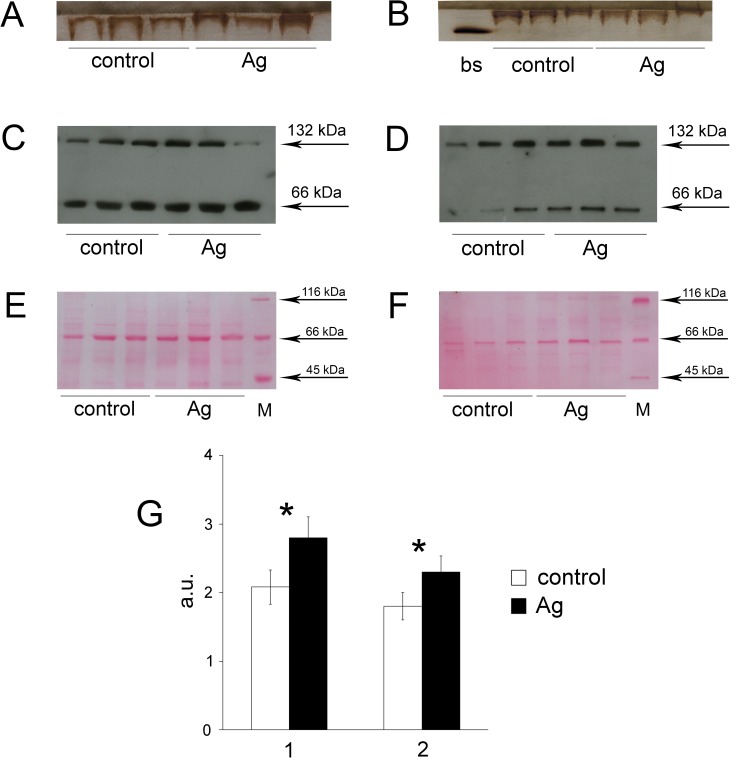
Oxidase Cp is formed in the cells of SAT. Aliquots containing 20 μg of protein per well of plasma membranes (**A**) and Golgi complex membranes (**B**) were processed by non-denaturing PAGE, and the gels were then stained with *o-*dianisidine during the night and treated with polyethylene glycol 6000 to minimize the gel square and enhance the intensity of the *o-*dianisidine zones (orange color). The first well in 4B (bs) is 1 μl of blood serum from a control rat. The data for three control rats and three Ag-rats are displayed. (**C**) Immunoblotting after 8% SDS-PAGE revealed the presence of Cp in the plasma membrane and (**D**) in the Golgi complex. The arrows indicate the molecular masses of polypeptides. (**E**) Load control (LC) for 4C (plasma membrane) and **(F)** the same for 4D (Golgi membranes). After transfer membranes were stained with PONCEAU S, scanned and 66-kDa zone were used as load control to calculated relative concentrations Cp polypeptides in Fig 4C and 4D. **(G)** The relative content of immunoreactive Cp polypeptides on the plasma membrane (1) and in the Golgi complex (2), both Cp fragments (66- and 132-kDa peptides) were accounted; a.u.;*: the difference was significant at the *P* < 0.05 level.

There are two principal questions in the present work: (1) does the Cp that is formed by SAT possess oxidase activity at the chronic Ag-fodder fed rats, and (2) is it secreted? To answer these questions, cellular fractions enriched with plasma or Golgi complex membranes were isolated from the SAT cells. The fractions were then analyzed electrophoretically (without SDS), and the gels were stained with *o*-dianisidine, a specific abiogenic chromogenic substrate for holo-Cp. The data in Fig 4A and 4B indicate that oxidase-positive Cp was present in these membranes from the Ag-rats. The mobility of serum Cp and GPI-Cp was different (Fig 4B). It is possible that electrophoretic separation was partially hampered by Triton X-100, which was used for membrane lysis. Triton X-100 promotes the formation of automicelles and micelles containing proteins with the GPI-anchor, preventing the focusing of the protein bands and reducing their mobility. These results cannot be compared with each other quantitatively because the gels were stained for a long period (overnight) and were then treated with polyethylene glycol 6000 to shrink the gels to enhance the bands. Nevertheless, the data definitely show that the SAT cells from the Ag-rats produced oxidase activity. There is a theoretical possibility that detectable oxidase activity belonged to hephaestin, an integral membrane protein from family of blue multicopper ferroxidase, which was able to oxidize *o*-dianisidine [31]. In rats, hephaestin is expressed at high levels throughout the small intestine and colon and at low levels in lung, spleen, placenta and embryo. It is not expresses in liver, kidney, brain, heart, skeletal muscle, and testis [32]. The data on hephaestin gene expression in adipose tissue are absent. Therefore, to identify the Cp at the plasma membrane and Golgi apparatus WB method was used. Both membrane fractions displayed the presence of immunoreactive Cp polypeptides, as revealed by antibodies to rat Cp (Fig 4C and 4D). Their molecular masses corresponded to full-length Cp (~130 kDa) and half of a Cp molecule (~65 kDa); the latter was formed due to limited Cp-specific proteolysis, which was typically observed on denaturing PAGE [33]. Statistical analysis of digital images showed that the relative content of immunoreactive Cp polypeptides was increased in both the plasma and Golgi complex membranes from the Ag-rats compared to the reference group (Fig 4E).

**Fig 5:**
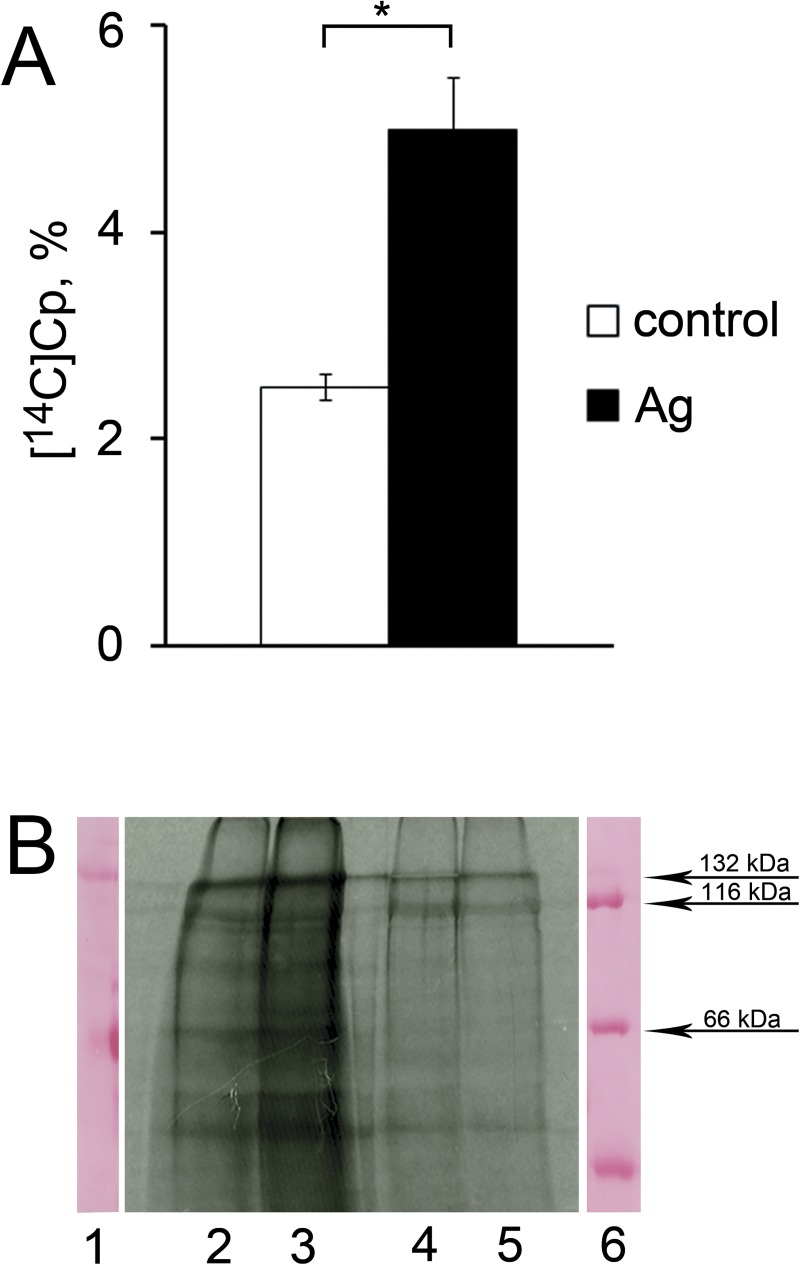
The SAT cells produce and secrete [^14^C]Cp. (**A**) The concentration of [^14^C]Cp, %, in the immunoprecipitates from the incubation medium. White bar–control; dark bar–Ag-rats. *: the difference was significant at the *P* < 0.05 level. (**B**) The autoradiograph of immunoprecipitates after 8% SDS-PAGE. Lanes: 1–1 μg preparation of rat Cp; 2 and 4 –control rat; 3 and 5 –Ag-rat; 2 and 3 –secreted proteins; 4 and 5 –protein extracted from membranes; 6 –protein markers.

In the next stage of the study, the ability of SAT to synthesize the sCp form was examined. The results presented in Fig 5A indicate that the slices of SAT tissue synthesized and secreted [^14^C]Cp under conditions of protein metabolic labeling. In the samples from the Ag-rats, the specific concentration of [^14^C]Cp was almost 2-fold higher than that in the reference samples. SDS-PAGE of the immunoprecipitates revealed that [^14^C]Cp was represented by the full-sized Cp molecule (~132 kDa) and a set of the characteristic Cp fragments from 19 to 116 kDa (Fig 5B) [33]. In these experiments, extracellular proteases also can contribute degradation of [^14^C]Cp. Additionally, [^14^C]Cp polypeptides were observed in the membrane fractions (Fig 5B). Their amounts were less than those in the medium; hence, sCp was accumulated in the medium during incubation.

## Discussion

Previously, we have shown that the holo-Cp level in the blood serum of rats that received the Ag-fodder for 1 month dropped to practically zero. However, the transcriptional activity of the *Cp* gene in the liver, the rate of hepatic Cp synthesis, and the content of immunoreactive Cp polypeptides in serum did not change [34,35]. The holo-Cp deficiency was caused by redox-inactive silver atoms because they occupied three Cys residues in the Cp active centers and replaced the copper ions of the type I enzyme that are required for redox catalysis [17,36]. We wanted to understand how the long-term, Ag-induced holo-CP deficit would affect mammalian development. Therefore, the holo-Cp deficit was initiated at birth and maintained throughout life. It was surprising that the blood holo-Cp level was approximately 50% of the normal value in rats that received the Ag-fodder for prolonged periods; thus, it was only decreased by a factor of two (Table 1), and the animals exhibited normal physiological development. However, in these rats, holo-Cp differed from hepatic Cp in its affinity for DEAE-Sepharose and lectins and in its antigenic properties. Moreover, the surgical isolation of the liver from the circulation with the subsequent [^14^C]amino acids pulse labeling of proteins *in vivo* indicated that there was a fraction of [^14^C]Cp that had a higher rate of secretion than hepatic Cp [17].

We hypothesized that the prolonged deficiency in holo-Cp in the circulation was compensated by the ectopic synthesis of holo-Cp in organ(s) in which the cells were not strongly affected by silver accumulation. Two criteria were used to find the organ-candidate that can synthesize and secrete holo-Cp into the circulation: the ability to accumulate silver ions and express the *Cp* gene. Of the organs under study, only skeletal muscles, IAT, and SAT did not accumulate silver (Fig 2). The Cp in circulation is believed to be primarily produced by the liver, and its main function is to regulate iron metabolism as a ferroxidase [37]. However, Cp has many various functions [38]. It has been proposed that Cp may function in copper transport, the oxidation of organic amines, the regulation of cellular iron levels, radical scavenging, neovascularization and possibly other processes. Moreover, it was shown that the sCp isoform was produced by different organs (brain, kidneys, spleen, leukocytes, and lungs) and that the rate of its synthesis was stimulated by inflammation [39] and suppressed by copper deficiency [39]. It is not known if the Cp synthesized by these organs contributes to the blood serum Cp level or plays some local roles in the extracellular spaces of the organs. Additionally, it is well known that the GPI-Cp isoform is expressed in many organs [40]. We searched for both Cp-mRNA isoforms produced by the *Cp* gene, and the testing was not strongly limited to the organs that did not accumulate silver. In all of the selected organs, Cp-mRNA isoforms coding for sCp and GPI-Cp were expressed (Fig 3A). These finding are in accord with the data cited above. It has previously been shown that sCp is synthesized in the SAT cells of obese patients and may enter the bloodstream [41]. The data on the synthesis of both splice isoforms of Cp-mRNA in the white adipose tissues are novel.

Of all tested organs, the *Cp* gene was only overexpressed in the SAT of the Ag-rats. This allowed us to think that SAT is the first candidate compensating holo-Cp deficiency in the bloodstream. Simultaneously expression level of GPI-Cp was increased. Additionally, the expression of the genes whose products participate in Cp/GPI-Cp metallation (*Ctr1* and *Atp7a/b*) was correspondingly increased in the SAT (Fig 3). The expression levels of both Cu-transporting ATPases were increased (Fig 3). It is known that ATP7A/B structure and domain topology, basic functions, cellular co-localization are similar; however, their specific functions are not identical [42]. While sCp co-expresses with ATP7B (in liver), GPI-Cp co-expresses with ATP7A (in brain) [43,44]. In SAT of Ag-rats, ATP7A and GPI-Cp mRNA’s relative levels were higher than ATP7B and sCp RNA’s. In the frame of this work it is difficult to find an explanation for this phenomenon.

The formation of oxidase-positive Cp and sCp in the SAT of the Ag-rats was important as a confirmation of our assumption. The oxidase Cp was detected in the plasma and Golgi complex membranes (Fig 4). The ability of SAT cells to synthesize secretory and membrane-anchored holo-Cp was confirmed in the metabolic labeling experiments (Fig 5). Thus, the hypothesis that SAT can compensate for the holo-Cp deficiency in the Ag-rats was verified and confirmed.

The prolonged Ag-diet did not affect the copper content in organs (Fig 2), despite the 15-fold excess of silver over copper in the fodder. The possible explanations for this effect are given below. Ag-fodder contains standard amounts of copper, and although CTR1 is mainly occupied by Ag(I), copper may still be transported to the cells by DMT1. Accordingly, the expression of the *Dmt1* gene is increased by a factor of two in the SAT cells of the Ag-rats (Fig 3). This assumption is supported by facts that in cultured human cells, derived from fetal kidney and umbilical vein endothelium, DMT1 compensates copper transport for CTR1 deficiency [45,46]. Although there are some contradicting data: it was shown that DMT1 is not required for the intestinal transport of copper using a mouse model lacking intestinal DMT1 [47]. In addition, the long-term maintenance of cellular copper homeostasis is compatible with transcriptional control of ionomics [48], the existence of a cellular system for copper recycling [20], and the slow development of copper deficiency following roux-en-y gastric bypass surgery [49]. In parallel to *Dmt1*, *Ctr1* gene expression was increased (Fig 3). Possibly, when CTR1 is occupied by silver and the copper deficiency developed, CTR1 up-regulation could operate as a mechanism that provides new CTR1 molecules to circumvent silver block and to allow Cu influx.

Adipose tissue is composed of various cell types, including adipocytes and other cells of the stromal vascular fraction, such as preadipocytes, blood cells, macrophages and endothelial cells. Adipocytes of the SAT and IAT are derived from different progenitor cells that exhibit different gene expression patterns [50]. However, the limitations of the present study do not allow us to suggest, what type of SAT cells synthesize sCp.

The data presented in this study provide definitive evidence that SAT cells produced Cp and the expression of the *Cp* gene at the transcriptional, translational and oxidase activity Cp formation levels were increased at the hepatic holo-Cp deficiency, and thus compensating for the lack of hepatic holo-Cp induced by the Ag-fodder. Until recently, white adipose tissue was only considered an energy storage organ. At present, it is viewed as an endocrine organ that secretes factors (adipokines, including Cp [41]) with autocrine, paracrine, and endocrine functions [50]. Perhaps, our work revealed new aspects of the physiological role of adipose tissue in mammalian copper homeostasis. It is possible that the blood Cp level during inflammation [51], tumor growth [17], pregnancy and lactation [52] is increased not only due to *Cp* gene activation in the liver, but also because the white fat cells produce holo-Cp more intensively.

Thus, subcutaneous fat can almost substitute for the liver with respect to the control of the holo-Cp level in blood. SAT may be a part of a system that controls the copper balance in the mammalian body, which may be used to elucidate the relationships between tumor growth, obesity and copper metabolism. Investigating the inter-organ regulation of copper metabolism in response to changing copper status may help us identify new antineoplastic approaches based on decreasing the bioavailable copper level [53].

## Abbreviations

Cp: ceruloplasmin
GPI-Cp: glycosylphosphatidylinositol anchored ceruloplasmin
sCp: soluble ceruloplasmin
IAT: internal adipose tissues
SAT: subcutaneous adipose tissues
